# Supplementary material for: The Bordetella type III secretion system effector BteA targets host eosinophil-epithelial signaling to promote IL-1Ra expression and persistence
Source: Commun Biol. 2025 Oct 20;8:1484. doi: 10.1038/s42003-025-08884-1 (PMC12537862; doi:10.1038/s42003-025-08884-1)
Supplement: Supplementary file 1 — Supplementary Information [file 42003_2025_8884_MOESM1_ESM.pdf]

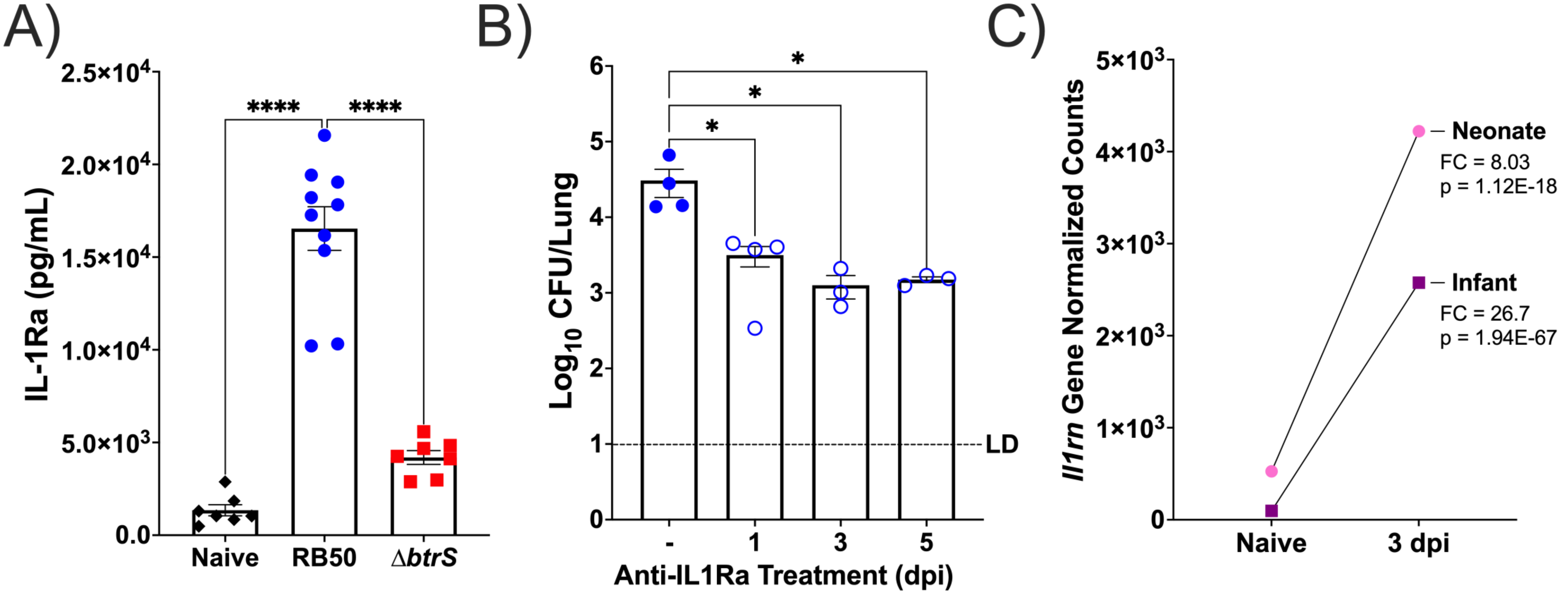

Supplementary Figure 1: IL-1Ra induction is conserved amongst classical Bordetellae and different age groups. BALB/c mice were intranasally challenged with RB50 (blue circles) or RB50Δ*btrS* (red squares). **(A)** Lung homogenate serum from naïve or infected mice were collected to perform measure IL-1Ra protein levels using ELISA. Each symbol represents one mouse, and the columns represent the mean ± SEM (n = 4-8). **(B)** Naïve or infected mice were intranasally inoculated with 30 µL of PBS containing 500 µg anti-IL-1Ra starting at 1, 3, or 5 dpi, and CFU were enumerated for each experimental group at 14 dpi (n = 4-10). **(C)** C57BL/6J neonate (pink circles) or infant (purple squares) mice were inoculated with PBS (naïve) or $6x10^9$ CFU/mL of Bp536. Lungs were collected for RNA sequencing at 3 dpi. Each symbol represents the mean raw counts normalized to PBS controls, with fold-change and its corresponding p-value of *Il1rn* gene expression (encoding IL-1Ra) denoted between naïve and infected age groups. Tukey's multiple comparisons One-Way ANOVA was used for statistical analysis of **(A-B)**. * = <0.05, **** = p<0.0001.

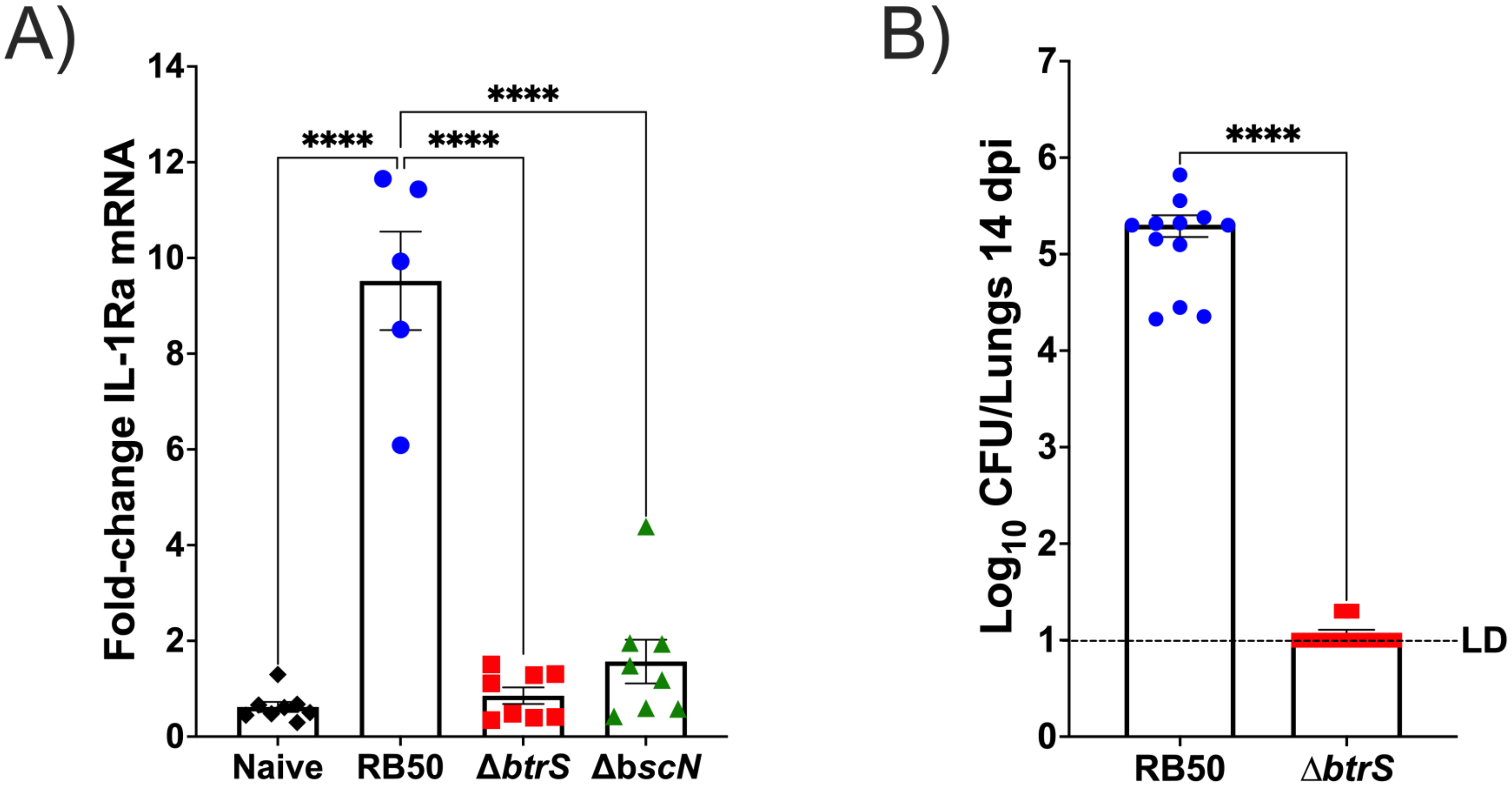

Supplementary Figure 2: The *Bordetella* spp. T3SS is required for IL-1Ra induction. (A) Naïve or infected mice were intranasally inoculated with 30 µL of PBS alone (black diamonds) or 10^6 CFU RB50, RB50Δ*btrS* (red squares), or RB50Δ*bscN* (green triangles), and lung RNA was extracted for qRT-PCR (n = 5-8). IL-1Ra gene expression was calculated compared to actin controls **(B)** Naïve or infected mice were intranasally inoculated with 30 µL of PBS alone (black diamonds) or 10^6 CFU RB50 (blue circles) or RB50Δ*btrS* (red squares). IL-1Ra expression is graphed as fold-change compared to uninfected controls and normalized to actin. Each symbol represents one plate well and the average of three technical replicates (n = 11-12). Tukey's One-Way ANOVA with multiple comparisons test was performed for statistical analysis. **** = p<0.0001.

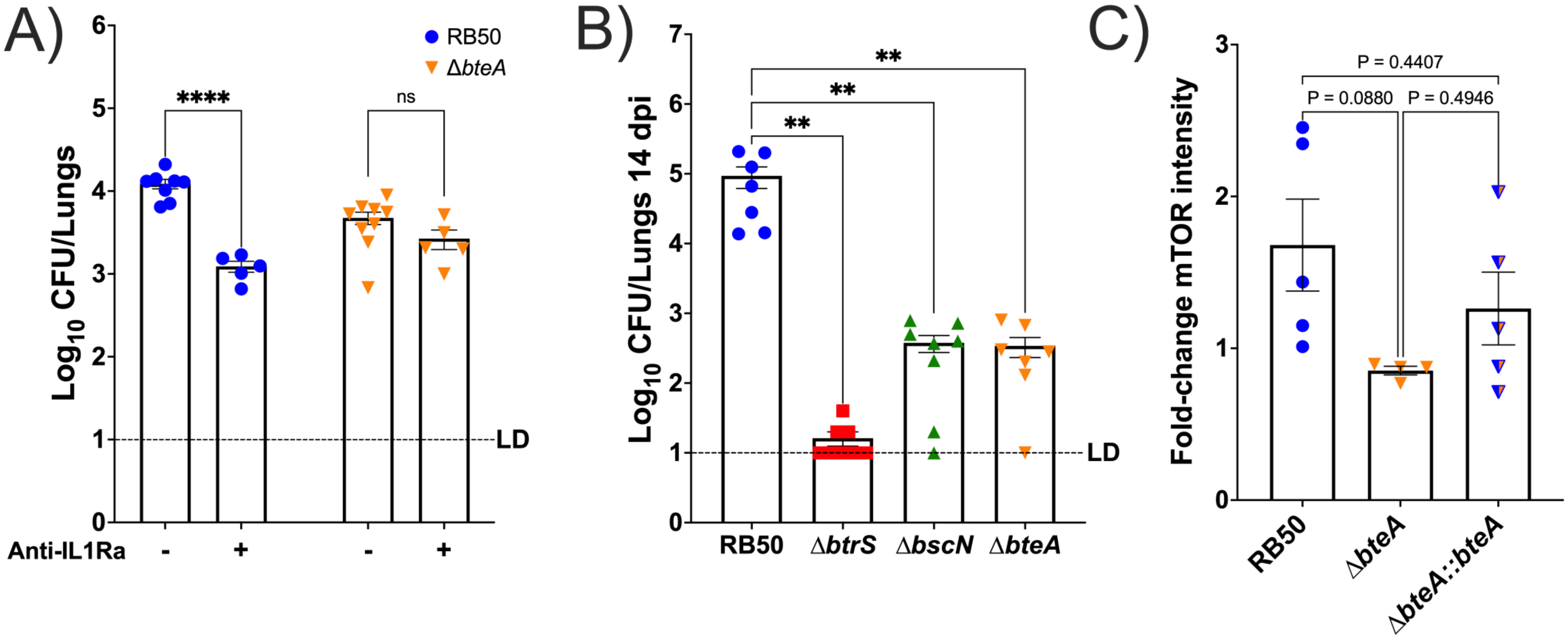

Supplementary Figure 3: IL-1Ra-mediated persistence of RB50 is BteA-dependent. (A) BALB/c mice were intranasally challenged with RB50 (blue circles) or RB50Δ*bteA* (orange inverted triangles) and administered anti-IL-1Ra or PBS from 5-14 dpi, then lung CFU were enumerated. Each symbol represents one mouse, with columns representing mean ± SEM (n = 5-8). **(B)** Lung CFU of BALB/c mice infected with RB50 (blue circles), RB50Δ*btrS* (red squares), RB50Δ*bscN* (green triangles), or RB50Δ*bteA* (orange inverted triangles) were enumerated at 14 dpi. Each symbol represents one mouse, with columns representing mean ± SEM (n = 7-8). **(C)** A549 cells were incubated RB50 (blue circles), RB50Δ*bteA* (orange inverted triangles), or RB50Δ*bteA::bteA* (blue/orange inverted triangles) at an MOI = 10. Cells were fixed and stained for phospho-mTOR at 2 hpi. Raw quantification values were normalized to mean naïve intensity for fold-change signal intensity. Each symbol represents one mouse, with columns representing mean ± SEM (n = 4-5). Tukey's Two-Way ANOVA was performed for **(A)**, and Tukey's One-Way ANOVA for **(B)** and **(C)**. ns = non-significant, ** = p<0.01, **** = p<0.0001.

Fig 1A: BALB CFU time course

	RB50												ΔbtrS											
0	420000	340000	44400	660000	558000	280000							102000	510000	456000	564000	516000	210000						
7	301000	539000	407000	2680000	3740000	126000	132000	292000	128000	260000			171000	86700	127000	172000	110000	372000	148000	110000	130000	330000	120000	410000
14	208000	199400	125000	210000	358000	142800	28000	664000	21200	22600	200000	240000	10	10	10	10	10	10	20	10	10	10	10	10
28	2000	1000	4000	1630	7720	1310							10	10	10	10	10	10	10	10	10	10	10	10

Fig 1B: IL-1Ra qRT time course

	Naive												RB50								ΔbtrS											
1	0.561	0.956	1.044	0.478									8.506	8.281	2.221	5.917					0.379	1.709	2.459	0.717								
7	0.478	1.296	1.226	0.501	0.608	0.294	0.659	0.674				0.448	9.928	11.434	9.131	8.541	11.654	6.088	8.506	13.596	0.410	0.394	0.345	1.286	1.111	1.308	0.477	1.509	1.304	0.726	0.294	1.296
14	0.054	1.946	0.644	1.356									0.077	0.100	0.952	0.640					1.425	0.096	0.673	0.554								
28	0.795	1.205	0.002	1.998									0.429	0.608	0.333	0.028					0.815	1.247	0.056	0.046								

Fig 1C: IL-1Ra ELISA time course

	Naive							RB50										ΔbtrS						
1	1041.9	274.74	260.65	1004.17	2681.19			7260.6	6570.4	7764.3	7738.2	6728	2180.6	5613.5	4491.9			2681.2	2564	1533.7	3145.7	3561	2982	
7	481.16	1036.5	847.17	1041.85	2877.55	1300.8	1847.2	10214	17822	19044	16170	18210	19424	21571	10317	17268	15356	4126.1	4677.7	4838.2	4252.6	5581	2887	2979
14	2858	1644.4	711.87	1886.33	3478.09			8873.5	5816.1	7865.4	3728.9	3133.1	4577.6	3312	4150.9			3192.4	4179.9	3511.2	2877.6	3487	3883	4166
28	612.89	1212	1283	1865.76	4252.56			3897.1	4066.1	8313.5	6861.8	5971.4	2564	1533.7				1390.3	1498.6	1644.4	1626.1	3192	4180	3511

Fig 1D: IL-1a ELISA D7

Naive	RB50	Δ*btrS*
1282.2	463.09	324.69
405.39	1395.2	220.83
1875.4	1263.4	174.28
772.99	2868.5	1939.2
811.54	699.43	1801.2
1569.6	430.01	364.69
629.81	372.74	521.81
727.58	1079.8	256.04

Fig 1E: IL-1b ELISA D7

Naive	RB50	Δ*btrS*
758.31	1597.9	444.89
347.72	1086.8	672.79
611.1	1547	790.92
1451.8	2794.8	908.17
590.63	1992.1	351
209.56	1983.9	252.74
517.23	1458.2	129.45
808.45	1863.8	
	1218.5	
	804.92	

Fig 2B: IL-1Ra IF Imaris time course

	Naive									
0	53	42	1	41	3	1	1			
1	53	42	1	41	3	1	1			
3	53	42	1	41	3	1	1			
7	53	42	1	41	3	1	1			

	RB50									
0	53	42	1	41	3	1	1			
1	104	19	46	37	42	39	40	94		
3	47	41	73	67	132	190	82	97		
7	50	79	207	224	56	87	38	33	90	44

	**Δ*btrS***									
0	53	42	1	41	3	1	1			
1	131	17	13	18	46	66	1	1		
3	31	22	51	1	40	101	*79**	84		
7	1	1	1	3	1	1	1	21		

	**Δ*bteA***								
0	53	42	1	41	3	1	1		
1	1	54	*39**	1	28	1	69	24	
3	24	86	1	18	6	12	9	1	3
7	1	1	1	2	54	20	69	35	

				Day 1								Day 3									Day 7						
Eosinc	0	5.2632	0	0	0	30.769	7.5	0	8.5106	0	4.1096	1.4925	3.0303	0	4.878	16.495	6	0	0	0	1.7857	0	15.789	0	34.091	66.667
Epithel	73.077	89.474	30.435	16.216	73.81	0	0	73.404	0	75.61	52.055	71.642	24.242	56.316	43.902	0	56	69.62	52.632	74.453	53.571	96.552	5.2632	76.667	0	0
Other	26.923	5.2632	69.565	83.784	26.19	69.231	92.5	26.596	91.489	24.39	43.836	26.866	72.727	43.684	51.22	83.505	38	30.38	47.368	25.547	44.643	3.4483	78.947	23.333	65.909	33.333

Fig 2C: A549 IL-1Ra qRT

Naive	RB50	Δ*btrS*
1.588	9.7	0.006
0.768	4.543	0.015
1.367	4.113	0.017
0.986	7.288	0.011
1.108	6.963	0.012
1.336	10.375	0.007

Fig 2D: bmEos IL-1Ra qRT

Naive	RB50	Δ*btrS*
0.599	3.451	1.113
0.911	3.869	1.389
0.242	4.343	1.267
0.63	8.335	0.331
0.819	6.456	0.607
0.943	11.042	0.817
	7.752	
	6.682	

Fig 3A: IL1RN KO D14 CFU

C57 RB50	*Il1rn*$^{-/-}$ RB50	C57 Δ*btrS*
208000	20	10
199400	330	20
125000	1	10
60000	1	1
49000	100	1
5200	30	1
5000		1

Fig 3B: IL-1Ra Supplementation CFU

RB50	RB50+	Δ*btrS*	Δ*btrS* +
1280	12890	20	240
3270	6440	10	160
490	7080	10	120
840	10240	10	180
740	2660	10	230
	2110	10	140
	1250	10	960
	3970	10	1560
		10	780
			940

Fig 3C: RB50 Anti-IL1Ra Tx D14 CFU

-	+	Isotype
2220	20	1820
3040	10	760
1800	10	830
2540	10	1100
1740	10	3150
1410	80	

Fig 3D: Bp536 Anti-IL1Ra Tx D14 CFU

-	+
7560	10
9600	20
2770	10
2020	1
	20

Fig 3E: Clinical Bp Anti-IL1Ra Tx D14 CFU

	Treated				Untreated				
BpI420	930	860	530	370	20	70	40	10	
BpJ021	700	280	570	680	30	60			
BpH787	420	280	770	620	60	70	130	40	140

Fig 4A: IL-1Ra qRT Neonate

Naive	D4	D8
0.5363	7.6821	12.4699
0.7917	7.931	6.2414
0.8784	6.0293	4.5674
2.6814	8.2078	3.9927

Fig 4B: Neonate Bp D4-8 CFU

D4	D8
82000000	280000000
66000000	276000000
144000000	166000000
54000000	1040000000

Fig 5A: IL-1Ra qRT in vivo T3SS

Naive	RB50	Δ*bscN*	Δ*bteA*	Δ*bteA*::*bteA*
0.816	3.883	0.748	0.580	3.000
0.976	2.849	1.033	1.172	4.360
0.319	5.041	0.717	0.422	4.840
0.709	1.973	0.739	0.596	2.490
0.319	4.171	0.923	0.550	1.270
0.835	9.928	0.513	0.493	1.150
0.833	6.088	0.650	0.840	1.970
	8.506		0.471	1.030

Fig 5B: IL-1Ra D7 ELISA in vivo

Naive	RB50	Δ*bscN*	Δ*bteA*	Δ*bteA*::*bteA*
2886.9	10317.37	4252.56	3487.21	9547.19
2979.18	5613.45	2681.19	3882.66	9198.43
1041.85	17268.49	2564.04	4166.26	9758.37
711.87	16169.53	1533.65	3959.28	4738.58
1886.33	16519.06		2905.4	
2180.63	18210.46		3692.23	
	13388.17			

Fig 5C: IL-1a D7 ELISA in vivo

Naive	RB50	Δ*btrS*	Δ*bscN*	Δ*bteA*
125.055	886.15	30.775	111.44	465.6
202.13	149.7	202.26	713.35	413.55
232.055	343.15	46.405	51.6	435.65
207.435	234.475	72.365		183.52
172.975	491.5	80.955		117.55
	87.65			312.565
	331.5			429.897
	290.6			

Fig 5D: IL-1b D7 ELISA in vivo

Naive	RB50	Δ*btrS*	Δ*bscN*	Δ*bteA*
798.945	1516.62	889.78	702	1370.28
543.395	695.44	1345.58	505.48	1104.82
773.475	1222.2	1581.84	258.9	1673.04
609.24	2903.62	1816.34		1484.72
402.46	1181.26			841.46
512.58	419.12			464.062
446.64	1034.46			576.339
	1616.9			

Fig 5E: A549 IL-1Ra qRT 2hpi

Naive	RB50	Δ*bscN*	Δ*bteA*	Δ*bteA*::*bteA*
1.1076	3.8026	0.8966	0.813	1.7466
1.3126	8.6676	0.4561	0.4909	1.4437
1.0099	1.7866	0.647	0.8329	1.7481
1.0247	3.7391	0.7223	0.9038	1.1879
1.0837	2.2067	0.6314	0.8329	1.6595
	4.5885			2.0149
				1.2285

Fig 5F: bmEos IL-1Ra qRT 2hpi

Naive	RB50	Δ*bscN*	Δ*bteA*	Δ*bteA*::*bteA*
0.599	3.451	0.331	0.727	1.735
0.911	3.869	0.607	0.721	1.996
0.242	4.343	0.423	0.662	2.133
0.63	8.335	1.729	0.64	1.691
0.819	6.456	0.72	0.105	1.891
0.943	11.042			2.367
0.817	7.752			
	6.682			

Fig 6A: A549 p-Akt ELISA

Naive	RB50	Δ*bteA*
1.8759	5.9346	1.656
1.112	4.0711	1.6729
1.064	4.7773	2.0249

Fig 6B: A549 Akt inhibitor qRT

	Naive				RB50						Δ*bteA*							
-	1.558	0.256	2.363	0.753	9.473	4.454	4.033	7.146	6.828	10.174	3.635	1.734	1.798	1.3	2.321	1.829	2.891	1.032
+	1.34	0.967	1.086		2.46	0.889	0.71	0.615			2.514	3.136	5.546	1.658	1.146	1.168	2.098	

Fig 7C: A549 p-mTOR ELISA

	Naive					RB50		Δ*bscN*		Δ*bteA*	
-	1.112	0.883	0.777	0.967	1.084	1.866	1.978	1.031	0.968	1.007	0.965
+	0.97	1.009	0.907	1.051	1.064	1.517	1.451	1.123	0.927	0.986	0.957

Fig 7D: A549 IL-1Ra Rapamycin qRT

	Naive			RB50							Δ*bteA*						
-	1.2	0.9709	0.8582	3.4755	6.237	4.6421	5.4853	10.1757	1.5538	2.2869	0.1141	0.7489	0.1315	0.768	0.3391	0.5318	0.0934
+	1.0086	0.9563	1.0368	0.2477	0.4722	0.187	1.2544	1.4424	2.0178		0.7414	0.5394	2.2869	1.6661	0.7991	0.6468	

Supp Figure 1A: D7 IL-1Ra ELISA

Naive	RB50	Δ*btrS*
481.16	10213.55	4126.11
1036.47	17822.13	4677.67
847.17	19043.98	4838.2
1041.85	16169.53	4252.56
2877.55	18210.46	5581.41
1300.8	19423.74	2886.9
1847.19	21570.66	2979.18
	10317.37	
	17268.49	
	15356.45	

Supp Figure 1B: Anti-IL-1Ra D1-3-5 CFU

-	1	3	5
13760	4500	2100	1700
14280	4030	1020	1540
28000	3740	660	1250
66400	340		

Supp Figure 1C: Bp536 C57 Neo vs Infant RNAseq

	Neonate	Infant
Naive	525.930042	96.4556122
3 dpi	4222.05437	2576.34472

Supp Figure 2A: BALBc T3SS IL-1Ra qRT

Naive	RB50	Δ*btrS*	Δ*bscN*
0.2939224	9.92837392	1.28551298	0.5799584
0.5012106	11.4337467	1.11101721	1.9568567
0.6077944	6.08791891	1.30781354	0.4215273
0.6589164	11.6539382	0.47708031	0.5956166
0.6743956	8.50589666	0.41	1.4841124
0.4478883		0.394	4.3894073
0.47778362		0.345	1.1823853
1.2961341		1.50893681	1.9379845

Supp Figure 2B: BALBc Lung CFU D14

RB50	Δ*btrS*	Δ*bscN*
208000	10	10
199400	10	20
125000	10	500
210000	10	400
358000	20	300
142800	10	800
28000	10	10
664000	10	200
21200	20	280
22600	10	130
200000	10	10
240000	10	

Supp Figure 3A: BALBc Anti-IL1Ra Tx D14 CFU bteA

	RB50								Δ*bteA*								
-	12890	6440	7080	10240	21000	14040	13240	13120	6000	4000	6420	8920	5290	3510	2410	680	5540
+	1700	1540	1250	1020	660				2065	1000	5155	2000	3145				

Supp Figure 3B: BALBc Lung D14 CFU

RB50	Δ*btrS*	Δ*bscN*	Δ*bteA*
208000	10	10	300
199400	10	20	800
125000	10	500	10
13760	10	400	670
14280	10	210	200
28000	20	720	280
66400	40	790	130
	20	370	

Supp Figure 3C: A549 mTOR IF quant

RB50	Δ*bteA*	Δ*bteA::bteA*
1.15010142	0.87423935	0.87829615
1.01101322	0.87444934	1.1277533
2.34722222	0.76851852	2.02777778
1.43640351	0.89473684	0.71052632
2.45454545	2.1969697	1.56439394
