# Supplementary material for: The Bordetella type III secretion system effector BteA targets host eosinophil-epithelial signaling to promote IL-1Ra expression and persistence
Source: Commun Biol. 2025 Oct 20;8:1484. doi: 10.1038/s42003-025-08884-1 (PMC12537862; doi:10.1038/s42003-025-08884-1)
Supplement: Supplementary file 2 — Reporting Summary [file 42003_2025_8884_MOESM2_ESM.pdf]

nature portfolio

Corresponding author(s): Monica Cartelle Gestal

Last updated by author(s): 5.Aug.2025

Reporting Summary

Nature Portfolio wishes to improve the reproducibility of the work that we publish. This form provides structure for consistency and transparency in reporting. For further information on Nature Portfolio policies, see our Editorial Policies and the Editorial Policy Checklist.

Statistics

For all statistical analyses, confirm that the following items are present in the figure legend, table legend, main text, or Methods section.

n/a	Confirmed	
☐	☒	The exact sample size (*n*) for each experimental group/condition, given as a discrete number and unit of measurement
☐	☒	A statement on whether measurements were taken from distinct samples or whether the same sample was measured repeatedly
☐	☒	The statistical test(s) used AND whether they are one- or two-sided<br>*Only common tests should be described solely by name; describe more complex techniques in the Methods section.*
☐	☒	A description of all covariates tested
☐	☒	A description of any assumptions or corrections, such as tests of normality and adjustment for multiple comparisons
☐	☒	A full description of the statistical parameters including central tendency (e.g. means) or other basic estimates (e.g. regression coefficient) AND variation (e.g. standard deviation) or associated estimates of uncertainty (e.g. confidence intervals)
☒	☐	For null hypothesis testing, the test statistic (e.g. *F*, *t*, *r*) with confidence intervals, effect sizes, degrees of freedom and *P* value noted<br>*Give P values as exact values whenever suitable.*
☒	☐	For Bayesian analysis, information on the choice of priors and Markov chain Monte Carlo settings
☒	☐	For hierarchical and complex designs, identification of the appropriate level for tests and full reporting of outcomes
☐	☒	Estimates of effect sizes (e.g. Cohen's *d*, Pearson's *r*), indicating how they were calculated

Our web collection on statistics for biologists contains articles on many of the points above.

Software and code

Policy information about availability of computer code

Data collection	Data collection was obtained for this study from animal experiments performed in a blinded manner.
Data analysis	Analysis was mostly performed with GraphPad

For manuscripts utilizing custom algorithms or software that are central to the research but not yet described in published literature, software must be made available to editors and reviewers. We strongly encourage code deposition in a community repository (e.g. GitHub). See the Nature Portfolio guidelines for submitting code & software for further information.

Data

Policy information about availability of data

All manuscripts must include a data availability statement. This statement should provide the following information, where applicable:

- Accession codes, unique identifiers, or web links for publicly available datasets
- A description of any restrictions on data availability
- For clinical datasets or third party data, please ensure that the statement adheres to our policy

Numerical data will be fully available upon request as well as made accessible via FigShare (DOI:10.6084/m9.figshare.29832140). Microscopy images will be available upon request.

Research involving human participants, their data, or biological material

Policy information about studies with human participants or human data. See also policy information about sex, gender (identity/presentation), and sexual orientation and race, ethnicity and racism.

Reporting on sex and gender	*Use the terms sex (biological attribute) and gender (shaped by social and cultural circumstances) carefully in order to avoid confusing both terms. Indicate if findings apply to only one sex or gender; describe whether sex and gender were considered in study design; whether sex and/or gender was determined based on self-reporting or assigned and methods used. Provide in the source data disaggregated sex and gender data, where this information has been collected, and if consent has been obtained for sharing of individual-level data; provide overall numbers in this Reporting Summary. Please state if this information has not been collected. Report sex- and gender-based analyses where performed, justify reasons for lack of sex- and gender-based analysis.*
Reporting on race, ethnicity, or other socially relevant groupings	*Please specify the socially constructed or socially relevant categorization variable(s) used in your manuscript and explain why they were used. Please note that such variables should not be used as proxies for other socially constructed/relevant variables (for example, race or ethnicity should not be used as a proxy for socioeconomic status). Provide clear definitions of the relevant terms used, how they were provided (by the participants/respondents, the researchers, or third parties), and the method(s) used to classify people into the different categories (e.g. self-report, census or administrative data, social media data, etc.) Please provide details about how you controlled for confounding variables in your analyses.*
Population characteristics	*Describe the covariate-relevant population characteristics of the human research participants (e.g. age, genotypic information, past and current diagnosis and treatment categories). If you filled out the behavioural & social sciences study design questions and have nothing to add here, write "See above."*
Recruitment	*Describe how participants were recruited. Outline any potential self-selection bias or other biases that may be present and how these are likely to impact results.*
Ethics oversight	*Identify the organization(s) that approved the study protocol.*

Note that full information on the approval of the study protocol must also be provided in the manuscript.

Field-specific reporting

Please select the one below that is the best fit for your research. If you are not sure, read the appropriate sections before making your selection.

☒ Life sciences ☐ Behavioural & social sciences ☐ Ecological, evolutionary & environmental sciences

For a reference copy of the document with all sections, see nature.com/documents/nr-reporting-summary-flat.pdf

Life sciences study design

All studies must disclose on these points even when the disclosure is negative.

Sample size	The numbers of mice that we have included in this study were calculated to obtain a power of 85-90% to in our analysis that allow us to detect statistical differences of SD 20%.
Data exclusions	All data collected in this study is included in the manuscript
Replication	All experiments were performed a minimum of 3 independent times including 2-3 technical replicates each time. The exact numbers are included in each result section
Randomization	All data was analyzed blinded and experiments were performed in a randomized manner. Groups were allocated randomly and blindly
Blinding	All experiments were performed blinded and data was unblinded after analysis. Groups were named A, B, C,... for experiments and analysis

Reporting for specific materials, systems and methods

We require information from authors about some types of materials, experimental systems and methods used in many studies. Here, indicate whether each material, system or method listed is relevant to your study. If you are not sure if a list item applies to your research, read the appropriate section before selecting a response.

Materials & experimental systems

n/a	Involved in the study
☐	☒ Antibodies
☐	☒ Eukaryotic cell lines
☒	☐ Palaeontology and archaeology
☐	☒ Animals and other organisms
☒	☐ Clinical data
☒	☐ Dual use research of concern
☒	☐ Plants

Methods

n/a	Involved in the study
☒	☐ ChIP-seq
☒	☐ Flow cytometry
☒	☐ MRI-based neuroimaging

Antibodies

Antibodies used

Describe all antibodies used in the study; as applicable, provide supplier name, catalog number, clone name, and lot number.

Validation

Describe the validation of each primary antibody for the species and application, noting any validation statements on the manufacturer's website, relevant citations, antibody profiles in online databases, or data provided in the manuscript.

Eukaryotic cell lines

Policy information about cell lines and Sex and Gender in Research

Cell line source(s)

Goat anti-mouse IL-1Ra detection an-tibody (Biotin) R&D Systems BAF480
Rat PE Conjugate Anti-mouse CD326 (Ep-CAM) Biolegend 118206
Rabbit Anti-Mouse PRG2 (Eosinophil Major Basic Protein) Abexxa Abx101775
Streptavidin AF488 Conjugate Invitrogen S11223
Goat Anti-Rabbit IgG AF647 Plus Highly Cross Absorbed Invitrogen A32733
Hoechst 33342 BD Biosciences 561908
Rabbit Phospho-mTOR Cell Signaling Technologies 5536S
Phospho-Akt (Ser473) Rabbit mAb Cell Signaling Technologies 4060S
Anti-E cadherin antibody [DECMA-1] – Intercellular Junction marker Abcam AB11512-1001
Donkey Anti-Rat AF594 Invitrogen S11223
Goat Anti-Rabbit IgG AF647 Invitrogen A32733
Hoechst 33342 BD Biosciences 561908
Sigma-Aldrich, Cat. #SRP6006anti-IL-1Ra94 (Leinco Technologies, Cat. #I-668) or Rat IgG2a isotype control (Leinco Technologies, Cat. #I-1177)

Authentication

Cell line were purchased from ATCC

Mycoplasma contamination

A549 epithelial cells were purchased from Sigma Aldrich (86012804-1VL). To ensure that the cells were not contaminated, tests for mycoplasma at each time that cells were pull from the freezer were performed. MycoAlert Mycoplasma Detection Kit from Lonza.

Commonly misidentified lines
(See ICLAC register)

Name any commonly misidentified cell lines used in the study and provide a rationale for their use.

Animals and other research organisms

Policy information about studies involving animals; ARRIVE guidelines recommended for reporting animal research, and Sex and Gender in Research

Laboratory animals

Mouse. All strains of mouse used are included on the manuscript including Balb/c, C57BL/6j, il1rn-/-

Wild animals

N/A

Reporting on sex

Results were first analyzed by sex, as no differences were found the data was pulled together

Field-collected samples

N/A

Ethics oversight

Our protocols are approved by the animal committee at LSU Health Science Center at Shreveport

Note that full information on the approval of the study protocol must also be provided in the manuscript.

Plants

Seed stocks	N/A
Novel plant genotypes	N/A
Authentication	N/A
